# COVID-19 hospitalizations and patients' age at admission: The neglected importance of data variability for containment policies

**DOI:** 10.3389/fpubh.2022.1002232

**Published:** 2022-11-30

**Authors:** Danila Azzolina, Rosanna Comoretto, Corrado Lanera, Paola Berchialla, Ileana Baldi, Dario Gregori

**Affiliations:** ^1^Unit of Biostatistics, Epidemiology and Public Health, Department of Cardiac, Thoracic, Vascular Sciences and Public Health, University of Padova, Padova, Italy; ^2^Department of Environmental and Preventive Science, University of Ferrara, Ferrara, Italy; ^3^Department of Public Health and Pediatrics, University of Turin, Turin, Italy; ^4^Department of Clinical and Biological Science, University of Torino, Torino, Italy

**Keywords:** COVID-19, overdispersion, prevention policies, hospitalizations, GDELT data

## Abstract

**Introduction:**

An excess in the daily fluctuation of COVID-19 in hospital admissions could cause uncertainty and delays in the implementation of care interventions. This study aims to characterize a possible source of extravariability in the number of hospitalizations for COVID-19 by considering age at admission as a potential explanatory factor. Age at hospitalization provides a clear idea of the epidemiological impact of the disease, as the elderly population is more at risk of severe COVID-19 outcomes. Administrative data for the Veneto region, Northern Italy from February 1, 2020, to November 20, 2021, were considered.

**Methods:**

An inferential approach based on quasi-likelihood estimates through the generalized estimation equation (GEE) Poisson link function was used to quantify the overdispersion. The daily variation in the number of hospitalizations in the Veneto region that lagged at 3, 7, 10, and 15 days was associated with the number of news items retrieved from Global Database of Events, Language, and Tone (GDELT) regarding containment interventions to determine whether the magnitude of the past variation in daily hospitalizations could impact the number of preventive policies.

**Results:**

This study demonstrated a significant increase in the pattern of hospitalizations for COVID-19 in Veneto beginning in December 2020. Age at admission affected the excess variability in the number of admissions. This effect increased as age increased. Specifically, the dispersion was significantly lower in people under 30 years of age. From an epidemiological point of view, controlling the overdispersion of hospitalizations and the variables characterizing this phenomenon is crucial. In this context, the policies should prevent the spread of the virus in particular in the elderly, as the uncontrolled diffusion in this age group would result in an extra variability in daily hospitalizations.

**Discussion:**

This study demonstrated that the overdispersion, together with the increase in hospitalizations, results in a lagged inflation of the containment policies. However, all these interventions represent strategies designed to contain a mechanism that has already been triggered. Further efforts should be directed toward preventive policies aimed at protecting the most fragile subjects, such as the elderly. Therefore, it is essential to implement containment strategies before the occurrence of potentially out-of-control situations, resulting in congestion in hospitals and health services.

## Introduction

Italy has been one of the first and most severely affected countries by the COVID-19 outbreak (1). During the early stages of the epidemic, the virus spread mainly in northern Italy (1). However, due to the full national lockdown declared in March 2020, the virus outbreak slowed down, reaching a bottom line in the summer (2). However, in October 2020, a second wave of the epidemic started in Italy (3), resulting again in considerable excess mortality (4, 5) and hospitalizations with extreme overloading of the health care system (6, 7). The vaccination campaign started at the end of 2020, reaching 78% coverage in the first year. On December 29, 2021, Italy reported 5,854,428 confirmed infections, 137,091 deaths, and 1885 hospitalizations in the intensive care unit (ICU) (8, 9). However, thanks to the vaccination campaign, the impact on the health care system remains small compared to 2020. For example, on November 30, 2020, there were 374 admissions to the ICU during the epidemic phase. One year later, the number of admissions was 683 despite the renewed resurgence of the spread of the epidemic at the end of the year (8).

COVID-19 does not affect the entire population exposed to virus contact in the same way; it is widely documented in the literature that the highest risk of severe disease, resulting in hospitalization and death, is among subjects older than 65 years (10). The Centers for Disease and Control Prevention (CDC) indicated a fourfold higher risk of hospitalization for COVID-19 for subjects between 65 and 75 years of age than for those between 18 and 29 years of age (10). Regarding mortality, an international study conducted in 16 countries indicated an 8.1 times higher mortality rate of COVID-19 for elderly individuals than for subjects between 55 and 64 years of age (11). An excess risk of death from COVID-19 and hospitalization in old age has also been reported in Italy (12, 13), documenting a median hospitalization age of 71 years for patients affected by COVID-19 (12, 14). For these reasons, the analysis of age trends at admission could help monitor the risk of older individuals, who are more susceptible to adverse outcomes, and quantify the possible impacts of the spread of the epidemic in other population sectors (15). However, quantifying the burden of the epidemic is not straightforward and one issue is represented by the variability in the number of hospital admissions. Excessive variability in daily admissions for COVID-19 could cause uncertainty and delays in implementing organizational decisions. From a statistical point of view, this excess of variability is under the vast concept of overdispersion, that is, an observed variability that overcomes what is foreseen under the model assumptions (16). However, the literature has poorly addressed overdispersion in hospitalization for COVID-19. Only a few studies have quantified the excess variability in COVID-19 infection (17, 18). In addition, these works have focused mainly on the spread of the disease (number of cases), which may be an indicator affected by the variability of time in testing policies, rather than hospitalizations (17, 18).

To our knowledge, no studies have addressed the extra variability pattern of COVID-19 infections according to the subject's characteristics. Many authors have focused on the ecological characterization of the impact of the epidemic on hospital facilities without evaluating its peculiarities using individual-level data (3). Other works evaluated overdispersion as a component of the spread of COVID-19 (17, 19, 20) without considering hospitalizations or assessing possible sources of extra variability in individual-level data.

The effects of an increase in daily fluctuations in healthcare demand have been poorly investigated in the literature. However, from an epidemiological point of view, a large daily hospitalization variability could make it difficult to plan the allocation of hospital beds and resources (21). For this reason, once a possible source of overdispersion has been identified, intervention policies could be tailored to active strategies in a preventive manner rather than as containment actions.

As highlighted above, the age at hospital admission is a leading predictor of disease severity. Therefore, this study aims to characterize a possible source of additional variability in the number of hospitalizations for COVID-19 as a function of the age at admission in Veneto region, Northern Italy from November 2020 to January 2021 using an inferential approach based on quasi-likelihood through the generalized estimation equation (GEE) model (22).

## Method

### Data

The Veneto region, Northern Italy administrative hospitalization regional registry data, including COVID-19 hospitalizations from February 1, 2020, to November 11, 2021, were considered for the analysis. The database includes information concerning (i) age at admission, (ii) date at admission, (iii) residence location, (iv) admission to the ICU, and (v) clinical status at admission.

### Statistical analysis

#### Data description

Hospitalization data were described by reporting continuous data as medians (I, III quartiles) and categorical data as percentages and absolute frequencies.

#### Characterization of the age of the hospitalization in COVID-19

The hospitalization age variable was described by reporting the age distribution according to the weeks of the pandemic. Data for each week are represented as box plots with medians, interquartile ranges, and outliers.

The distribution of the COVID-19 hospitalization number was reported in general and by age to describe the impact of the disease on hospital facilities.

Lower outlier ages (values <1.5 times the first quartiles) were identified in the database. The impact of the pandemic evolution time (weeks) on the extremely lower hospitalization ages were evaluated with a Poisson generalized linear model. The IRR (incidence rate ratio) with 95% confidence interval (CI) was reported.

#### Model overdispersion in hospitalization

The number of hospitalizations was modeled using a GEE approach with a Poisson link function. In our research, we were interested in characterizing age as a possible factor impacting the overdispersion of daily hospitalizations. The Generalized Estimating Equations (GEE) have been considered for the computation because it lies on a semi-parametric approach. The GEE method is based on the definition of a model for the mean and variance of the outcome and a working correlation structure, but it is not required to define the distribution of the endpoint (22).

A clustering structure was considered to account for outcome correlation within the same Local Health District (LHD). A natural spline was used for the time effect. The scale parameter, [[Inline Image]], is the component accounting for overdispersion and represents the random variability in the mean of the Poisson distribution (22). Natural splines were used by assuming linear constraints at the boundaries; the smoothing approach has also been considered elsewhere for COVID-19 epidemiological research (23). In addition to its computational convenience, this approach is characterized by compact support, providing a particularly sensible fit of the incidence rates at the extremes of the study period, specifically at the end of that period. This is particularly convenient for epidemiological surveillance purposes (24). The optimal number of approximation degrees was achieved by minimizing the mean squared error (MSE).

The GEE model included age as the scale covariate to parameterize its effect on the overdispersion in COVID-19 hospitalizations. In addition, age class was also considered a covariate for the number of hospitalizations in GEE modeling.

The GEE overdispersion parameter was estimated under various assumptions regarding the correlation structure: exchangeable, autoregressive, and unstructured (25). The goodness-of-fit for the different model parameterizations was evaluated by considering the correlated information criterion (CIC). We considered the CIC also to choose among different model formulations; the smaller value of the CIC criterion leads to a better model structure (26). The CIC compares the sandwich and naive covariance estimators and demonstrates improved performance compared to the quasi-likelihood information criterion (QIC) (27). The diagnosis of residuals has been also reported in the Supplementary material.

Model predictions and effects were reported; the IRR behavior is shown in weeks. The coefficients of the nonspline terms are the first derivatives (slopes) of their contributions to the model (28). The spline causes these effective coefficients to vary with the weeks of the pandemic. The varying IRR effect was reported by computing the first derivatives of predictions according to weeks.

The effects of time on the number of hospitalizations and of age on the overdispersion parameter were reported for the model that minimized the CIC criterion.

Other details concerning the method have been reported in the Supplementary material.

### Evaluation of public health policies

Possible epidemiological impacts of the COVID-19 hospitalization overdispersion phenomenon were evaluated considering the association between past variations in hospitalizations and the number of news items (internet, newspaper, etc.) indicating the activation of pandemic prevention policies; the inflation in the number of preventive initiatives could be an indicator of difficulties in adapting the health care system (29).

Data on the number of news items related to public prevention policies designed to contain the effects of COVID-19 were identified from the GDELT database (30). The GDELT Global Knowledge Graph database contains categorized news from all countries of the world, updated every 15 min (30). The news data from February 2, 2020, to November 7, 2021, were downloaded. Records concerning the Veneto region in Italy were identified in the “Location” field of the database.

The news concerning COVID-19 policies was identified by selecting the news topics identified by the key search (“coronav” OR “COVID”) AND (“policy”) AND (“health”) AND (“disaster response”).

The daily variation in the number of hospitalizations in the Veneto region that lagged at 3, 7, 10, and 15 days was associated with the number of news items related to containment interventions to determine whether the magnitude of the past variation in daily hospitalizations could impact the number of preventive policies implemented.

A least-squares estimation with the restricted cubic spline on the number of hospitalizations was performed to account for nonlinearities.

The increasing number of prevention policies can be considered a proxy for the uncertainty about the adoption of the most appropriate prevention measures (29).

## Results

### Sample description

Sixty-one thousand twenty-two hospitalizations occurred in Veneto from February 1, 2020, to November 11, 2021. Hospitalizations are referred to one of nine LHDs in the Veneto region. The median age at admission was 75 years; the highest percentage of hospitalized patients was in the age class 75–85. Age classes below 50 years were underrepresented among COVID-19 hospitalizations. The largest part of the sample (60%) comprised males and Italian people (93%).

Sixteen percent of patients were affected by other comorbidities, mainly cardiovascular diseases (12%). Seventeen percent of hospital admissions involved at least one ICU transition (Table 1). ICU transitions are defined in the database as the number of hospitalized patients with COVID-19 who have had at least one ICU visit during their hospital stay.

**Table 1:** Continuous data are reported as median (I, III quartiles); categorical data are reported as percentages and absolute frequencies.

	**(*N* = 61,022)**
Age classes: < 30	2% (1,107)
(30, 40]	2% (1,503)
(40, 50]	6% (3,932)
(50, 65]	23% (14,315)
(65, 75]	23% (14,319)
(75, 85]	26% (16,135)
>85	16% (9,711)
Gender: Female	40% (24,442)
Male	60% (36,579)
Nationality: Foreign	7% (3,983)
Italian	93% (56,132)
Death: no	77% (46,955)
Yes	23% (14,067)
ICU admission: No	83% (20,649)
Yes	17% (4,177)
Hospitalization to death [days]	3/7/13
Hospitalization to healing [days]	4/8/15
Local health district: ULSS n. 1 Dolomiti	5% (2,817)
ULSS n. 2 marca trevigiana	20% (11,902)
ULSS n. 3 serenissima	13% (7,576)
ULSS n. 4 veneto orientale	3% (1,771)
ULSS n. 5 polesana	4% (2,596)
ULSS n. 6 euganea	19% (11,439)
ULSS n. 7 pedemontana	7% (4,332)
ULSS n. 8 berica	8% (5,072)
ULSS n. 9 scaligera	21% (12,435)

The median duration from hospitalization to death was 7 days, and that from hospitalization to healing was 8 days (Table 1).

### COVID-19 hospitalization ages

**Figure 1:**
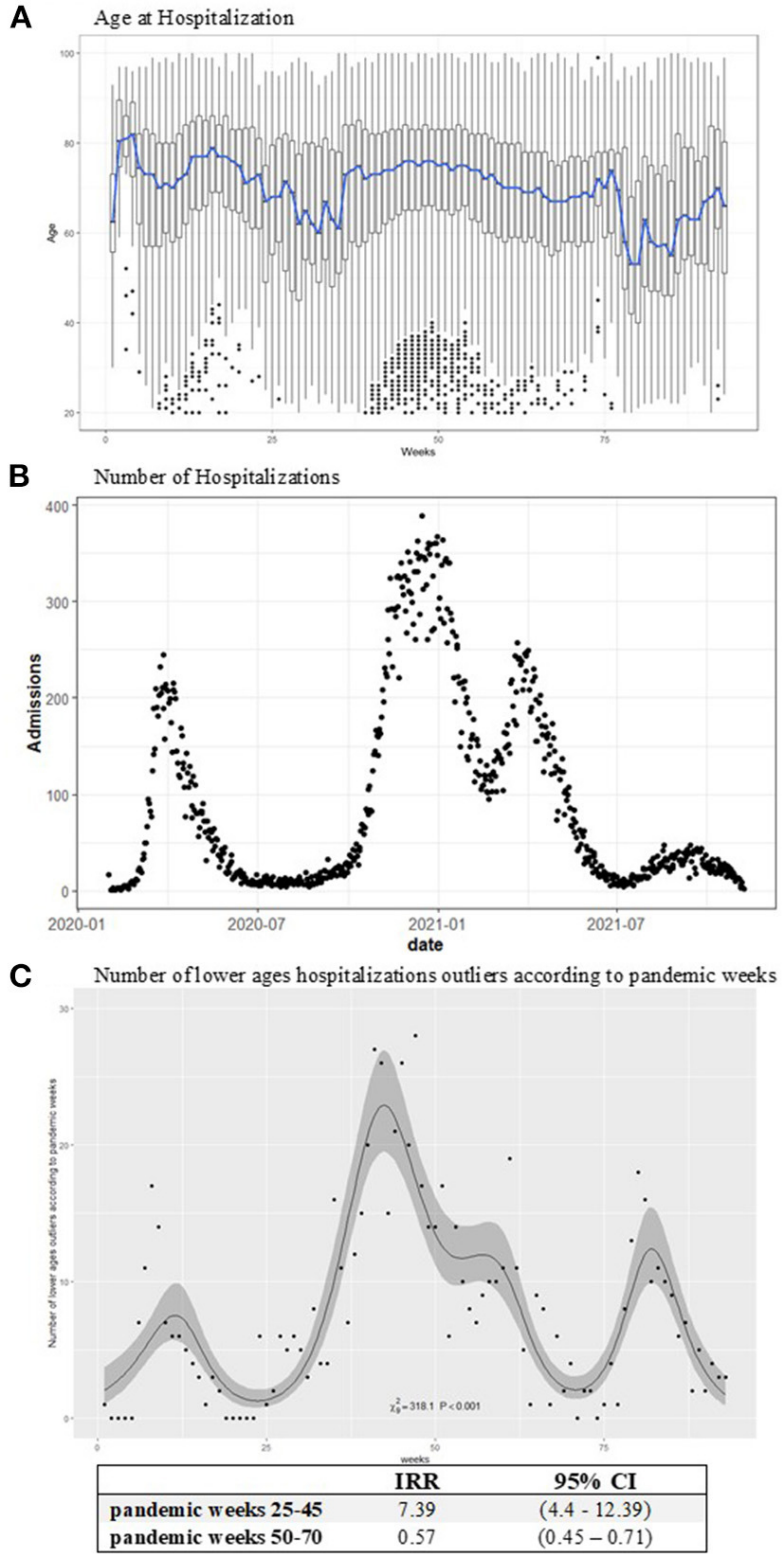
Age at COVID-19 hospitalization boxplots **(A)**, the blue line represents the mean age by week. The number of hospitalizations according to days **(B)**. **(C)** Reports the observed (points) and estimated (line) number of lower ages outliers with the IRR effects according to pandemic weeks with 95% confidence intervals. The models have been estimated on the weekly data. An increasing incidence in the number of lower ages hospitalizations is observed in the expansive phase of the pandemic: 25–45 weeks IRR (95% CI) = 7.39 (4.4–12.39).

Weekly box plots of hospitalization ages showed a stable mean of 75 years. A slight decrease in hospitalization age to 60 years was observed close to epidemic recovery periods during the summers of 2020 and 2021. Moreover, a rising pattern in the number of outliers for hospitalizations among younger ages was highlighted during the second spreading phase of the pandemic from November 2020 to January 2021 (Figure 1A). A similar phenomenon could be was noted considering the single LHD in Supplementary Figure S1. An increasing effect on the number of hospitalizations among younger ages was observed between November and January (25–45 pandemic weeks), with an IRR of 7.39 (95% CI = 4.4–12.39). Instead, a decrease in the IRR was evidenced between the 50th and 70th weeks of the pandemic (Figure 1C).

The number of hospitalizations raised in the second wave of the epidemic, beginning the first days of December with an increase in daily fluctuations in admissions. This increase was followed by an epidemic reappraisal period from May through October 2021. Then, the epidemic again had an impact on hospitalizations, although to a lesser extent than in the previous year, as of the end of October 2021 (Figure 1B). This trend was also confirmed by looking at the numbers of hospitalizations by age class. The greatest fluctuations in hospital admissions were observed among the elderly (>65 years), especially in the second and more widespread phase of the pandemic (Supplementary Figure S2).

### Model for overdispersion

The results of the GEE model showed a similar excess of dispersion in the hospitalization data across the different parameters of the working correlation matrix. The Auto Regressive (AR) correlation structure minimized both the QIC and the CIC criteria (Table 2) and thus was selected as the reference parameterization. Noticeably, considering higher lags did not improve the goodness-of-fit. Moreover, the residual analysis is deemed to be satisfactory for the model diagnostic (Supplementary Figure S3).

**Table 2:** Estimated GEE overdispersion (ϕ) with SE (standard error) according to the working correlation matrix.

**Working correlation**	**ϕ**	**CIC**
AR	**21 (5)**	**24**
Exchangeable	19 (4)	32
Unstructured	17 (3)	107

The goodness of fit for the different parametrizations has been evaluated by CIC criterion.

The parameterization that optimizes the criterion was highlighted in bold.

Table 3 shows the effect of the model on the number of admissions and the overdispersion of the AR parameterization.

**Table 3:** GEE estimates.

**Panel A**. Model predictions according to weeks with 95% confidence bounds.	
**Panel B**. Effective IRR (Incidence Rate Ratio) according to weeks. The varying IRR has been calculated by computing the first derivatives of predictions	
numerically and dividing the successive differences in predicted values by the successive variation in time unit (week).	
**Panel C**. The age effect on overdispersion has been reported (ϕ). The AR correlation structure minimizing the QIC and CIC criteria has been considered.	
The standard errors and p-values have also been reported.	
Panel A Model predictions	Panel B Effective IRR
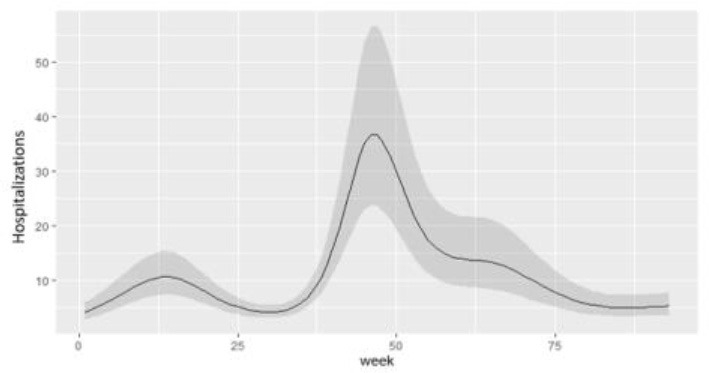	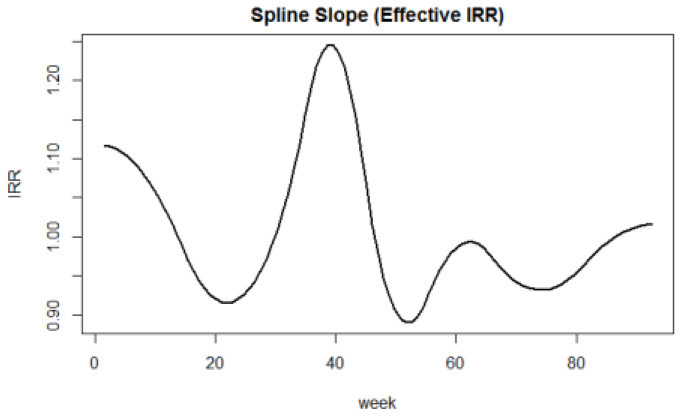
Panel C GEE Hospitalization Model and Overdispersion Estimates	
	**Estimate** ^**^	**Standard errors**	***P*** **-value**
Hospitalization covariate^*^			
Age Classes: < 30	ref		
30-40	1.522	0.054	< 0.001
40-50	2.745	0.053	< 0.001
50-60	6.108	0.085	< 0.001
60-70	7.285	0.067	< 0.001
70-80	7.328	0.071	< 0.001
>80	5.457	0.065	< 0.001
**Overdispersion covariates** ϕ^**^			
Age Classes: < 30	ref		
30–40	4.872	2.251	0.03
40–50	42.68	11.55	< 0.001
50–60	544.9	156.5	< 0.001
60–70	587.9	190.1	< 0.001
70–80	707.8	226.4	< 0.001
>80	249.6	77.58	< 0.001
**Correlation parameter**			
**α**	0.99	0.001	< 0.001

* The estimate expresses the IRR (Incidence Rate Ratio) effect on the Hospitalization outcome.

** The estimate represents an identity effect (multiplicative increase) on the overdispersion parameter.

The prediction of hospitalizations for LHD showed a lower peak during the first phase of the pandemic through the summer of 2020 to week 25; a higher peak in hospital admissions was evidenced throughout the fall and winter of 2021, from week 30 to week 60 of the epidemic (Table 3, Panel A).

The estimated effect of the IRR exceeded the growth limit of 1 in the first phase of the pandemic through April 2020. During the second wave, the incidence of hospitalization growth exceeded this limit in the December 2020 through February 2021 period (weeks 30–50). A further increase in hospitalizations was estimated from late October 2021 but did not exceed the growth limit of an IRR unit (Table 3, Panel B).

Age at admission affected the excess variability in hospitalizations, and this effect increased as age increased. Specifically, compared to subjects with an age <30, for the age group between 30 and 40 years, the dispersion increased four times; for ages between 40 and 50 years, the variability increased 43 times, until it achieved a maximum increment for ages between 70 and 80 years (Table 3, Panel C).

### Impact of overdispersion on public health models

**Figure 2:**
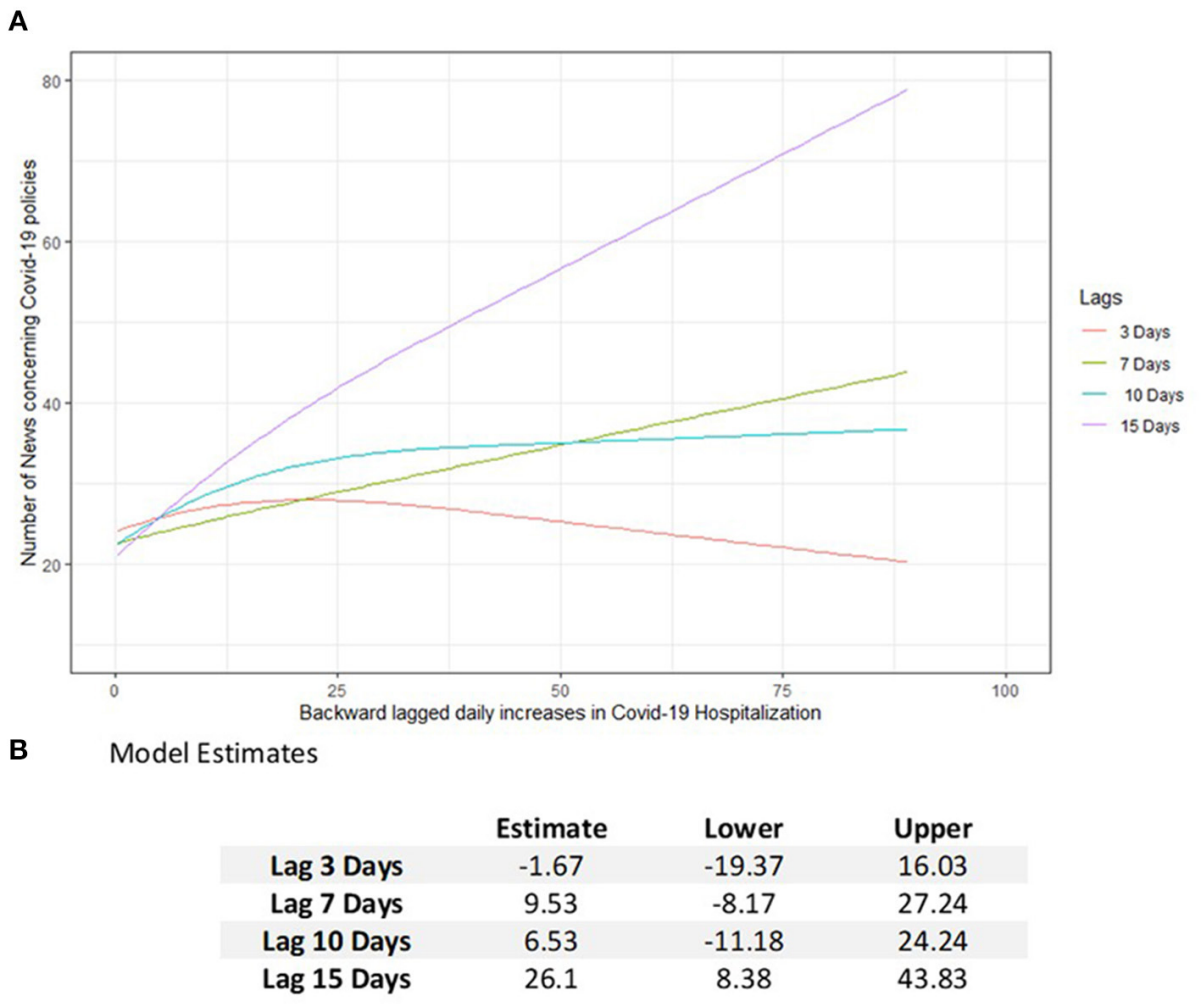
Estimated number of news about the COVID-19 policies **(A)** in Veneto according to daily increases in the number of hospitalizations lagged backward by 3, 7, 10, and 15 days. **(B)** Model estimation table reporting the increasing effects in the number of COVID-19 policies with 95% CI subsequently to an increase of hospitalizations in the past days from 10 to 50.

Fifteen thousand two hundred and seventy-four COVID-19 policy news records concerning the Veneto region were identified in the considered period. The estimated model revealed a significant increase in the number of news reports on pandemic policies (26.1, 95% CI = 8.38–43.83) and a subsequent increase in daily hospitalizations from 10 to 50 (Figure 2). Furthermore, a significant interaction effect between hospitalizations and backward lags was observed (*p* < 0.001).

## Discussion

This study showed a significant long-term increase in the number of hospitalizations for COVID-19 in Veneto from December 2020 to February 2021. During this period, the Veneto region, in Northern Italy experienced less severe containment measures because its health care system was effective at reducing the stress of hospital facilities (31). Therefore, the virus spread more and caused a prolonged increase in hospitalizations during the second phase of the epidemic compared to other regions.

This study found an excess of variability in the pattern of daily hospitalizations in the Veneto region during the second phase of the epidemic. The literature addresses overdispersion, mainly referring to the spread pattern of COVID-19 instead of hospitalizations. A study conducted in South Korea explored how the risks of coronavirus infection interacted between age groups through modeling the overdispersion parameter by estimating a flexible generalized Poisson model fitted with the Bayesian approach (32). Another study evaluated the level of overdispersion in COVID-19 transmission using a mathematical model defined by a transmission rate and an overdispersion parameter of a negative binomial branching process (17). Another study carried out in Indonesia suggested that overdispersion events played a key role in the early stage of the outbreak, where a large number of infected individuals were responsible for a large number of COVID-19 transmissions (33).

This work, instead, highlights an additional source of variability (overdispersion) in hospital admissions, especially during the main expansion phases of the epidemic. From a statistical point of view, as already assessed by other authors, when an evident source of overdispersion is estimated, further sources of extra-variability should be examined. Hence, the model should be adjusted accordingly to account for such factors affecting this extra-variability (34). This work focused on the role of overdispersion in the number of hospitalizations, explaining the pattern of the phenomenon according to different age groups.

The increasing instability in the daily trend of hospital admissions was evident in subjects with 65 years of age or older. As a consequence, the daily number of hospitalizations for COVID-19 among people under 50 years of age was lower and less variable. Moreover, in line with the literature, the mean age at admission was steady at ~75 years also in this study (11).

In autumn 2021, again, a greater increase in hospitalizations was evident. Furthermore, mass immunization, which first affected frail and elderly people, may have contained the effects of the pandemic on hospitalizations (35) despite the marked spread of the virus that was evidenced at the end of October 2021.

### Public health impact of overdispersion

The lesson learned from this pandemic is that a widespread circulation of the virus, primarily involving elderly and frail individuals, would result not only in a serious impact on the health care system but also in a source of additional variability in hospitalizations that would make it difficult to plan prompt health care policies. Our model showed that a change from 10 to 50 hospitalizations occurring in the previous 15 days could increase the production of interventions aimed at limiting the effects of COVID-19 on health care facilities. An uncontrolled increase in policies could be interpreted as a proxy for incurring difficulties in managing the phenomenon of overdispersion and its impact on the health care system.

Intervention policies should consider these issues by tailoring their efforts to allocate hospital resources specifically to elderly individuals who are more fragile and more at risk for severe COVID-19 (hospitalization, ICU admission, and death). Furthermore, health care facilities must be aware of these fluctuations and have to adapt the available resources, in terms of accommodations and staffing, to these particularly at-risk groups.

### Public health policy implications

This study demonstrated that to prevent the uncontrolled effects of fluctuation in the daily hospital admission for COVID-19, it would be useful to limit the disease spreading especially among elderly subjects.

Mass vaccination represents the most important intervention. his campaign has been launched in Italy on 27 December 2020, as in other European countries, and continues to reduce COVID-19 hospitalization, mortality, and severe disease outcome, by protecting also the healthcare system overloads (36). In particular, the Center for Disease and Control Prevention estimates that people aged more than 65 years fully vaccinated with an mRNA COVID-19 vaccine had a 94% reduction in risk of hospitalizations, and the vaccine is 64% less effective in subjects partially vaccinated (37).

Within this general framework, communication policies promoting vaccination program completion, especially among the elderly, are strongly recommended; clear information should be provided concerning the benefits of the primary vaccine administration and boosters, especially for fragile population groups (38).

Another possible intervention for limiting the disease spreading among people aged more than 65 years, is the adoption of an efficient contact tracing policy for the control of COVID-19 diffusion. The timely identification and management of COVID-19 cases could promptly identify secondary infections by interrupting the subsequent transmission process (39).

It is well recognized, in the medical literature, that a reliable test for diagnosing COVID-19 is the reverse transcription-PCR (RT-PCR) test which is highly effective in detecting the infection in subjects who are symptomatic but is less effective for pre-symptomatic patients (40). As frequent testing could be an expensive procedure, the PCR test is generally performed only on symptomatic cases (41). For this reason, digital contact tracing systems have been proposed in the literature as a cost-effective monitoring policy to control COVID-19 spreading, especially among the elderly and long-term hospitalized patients (41). However, in Italy and in many other countries, contact tracing through smartphone apps and wearable devices has played a marginal role in the management of COVID-19, even if the literature demonstrated that electronic tracing facilities can be effective at controlling new diseases spreading without quarantining large groups of the exposed population (42).

In this framework, educational support policies facilitating the use of web applications and devices among older people could be useful to avoid the so-called “contact tracing paradox:” these electronic devices and apps would have a greater epidemiologic benefit for the elderly, who, at the same time, however, represents the part of the population less inclined to technology and most suspicious of sharing personal data (43).

All the aforementioned policies constitute a preventive mechanism to control the spreading of the virus within the most at-risk population group: the elderly people. In contrast, as our analysis showed, the many policies adopted in Veneto and other Northern Italian regions during the early months of 2021 were mainly containment policies. Therefore, these policies were aimed at quarantine, contact limitation, and promotion of the use of personal protective equipment, and acted as a kind of ex-post control over a process (the pandemic) that had already begun (44). However, especially in the elderly, these interventions aimed primarily at limiting contacts (resulting in social isolation) have led to psychological distress issues, exacerbating, in several cases, already critical pathological situations (45). Therefore, the challenge for living with the virus could be to promote vaccination and efficient monitoring of the spread of the disease, protecting older individuals without depriving them of basic relational needs.

### Study limitations

Quantification and characterization of overdispersion as an additional source of uncertainty regarding a disease spreading requires continuous and up-to-date surveillance; the phenomenon should be described at the micro territorial level. It is known that epidemics can be concentrated in specific geographically located outbreaks (46); therefore, the structure of LHDs is too broad to characterize the phenomenon of capillary action on the territory. Further efforts are needed to monitor sources of overdispersion at the micro geographical levels by adjusting for other factors (events, population movements, traffic) that could impact on diseases (47).

Additional predictors that could affect the number of hospitalizations and overdispersion are comorbidities. This information was not considered in our analysis because it was incomplete and underreported, especially during the first phase of the pandemic. However, the literature has extensively demonstrated that patient age is the leading predictor of the most important outcomes of COVID-19 (48).

Moreover, the leading purpose of the manuscript is to identify the characteristics affecting the extra variability of hospitalizations, especially during the spreading phase of the pandemic. The estimation model is not defined for forecasting or predictive reasons; other research developments are needed to define a forecasting tool for the COVID-19 hospitalizations.

## Final remarks

Overdispersion in COVID-19 hospital admissions is an observed phenomenon, especially during the phases of the epidemic spread. Elderly patients are the subjects most affected by this source of uncertainty, which could lead to huge efforts for emergency services planning during the pandemic. However, this study shows that, regardless of whether the epidemic is particularly widespread in the territory, even the youngest age groups can lead to an overload in hospital activities, also acting as a vector of contagion to the most vulnerable population groups.

## Data availability statement

The datasets presented in this article are not readily available because data would be made available upon reasonable request to the authors. Requests to access the datasets should be directed to dario.gregori@unipd.

## Ethics statement

Ethical review and approval was not required for the study on human participants in accordance with the local legislation and institutional requirements. The patients/participants provided their written informed consent to participate in this study.

## Author contributions

DA: original draft preparation and statistical analysis. RC, IB, CL, and PB: writing–review and editing. IB, DA, DG, and PB: methodology. DG: supervision. All authors contributed to the article and approved the submitted version.

## Conflict of interest

The authors declare that the research was conducted in the absence of any commercial or financial relationships that could be construed as a potential conflict of interest.

## Publisher's note

All claims expressed in this article are solely those of the authors and do not necessarily represent those of their affiliated organizations, or those of the publisher, the editors and the reviewers. Any product that may be evaluated in this article, or claim that may be made by its manufacturer, is not guaranteed or endorsed by the publisher.
