# Supplementary material for: COVID-19 hospitalizations and patients' age at admission: The neglected importance of data variability for containment policies
Source: Front Public Health. 2022 Nov 30;10:1002232. doi: 10.3389/fpubh.2022.1002232 (PMC9748343; doi:10.3389/fpubh.2022.1002232)
Supplement: Supplementary file 1 [file Data_Sheet_1.docx]

Supplementary Material

# Model overdispersion via the GEE approach

Liang and Zeger (1) introduced the GEE method for the regression of correlated data. This approach extended the quasi-likelihood estimation method to the clustered data.

Assuming a sample of observations *i = 1, …, K* independent and multivariate, $Y_{i}=\left( Y_{i1},\ldots,Y_{\mathrm{it}},\ldots,Y_{in_{i}} \right), where$i may represent a cluster with $n_{i}$ observations. The expectations $E\left( Y_{\mathrm{it}} \right)=\mu_{\mathrm{it}}$are related to the p dimensional regressor vector $x_{\mathrm{it}}$ via the mean-link function g

$$\begin{aligned} g\left( \mu_{\mathrm{it}} \right)=x_{\mathrm{it}}^{\top}\beta\\ VAR\left( Y_{\mathrm{it}} \right)=\phi a_{\mathrm{it}} \end{aligned}$$

The $\phi$ parameter is the scale dispersion parameter and $a_{it}=a\left( \mu_{it} \right)$ is the variance function. The working correlation matrix $R_{i}(\alpha)$depending on the *m* parameters $m.$ The covariance matrix $V_{i}$ of $Y_{\mathrm{it}}$

Depends on the scale overdispersion parameter $\phi$ and a diagonal matrix $A_{i}$ with generic entry $a_{it}$.

$$V_{i}=\phi A_{i}^{1/2}R_{i}(\alpha)A_{i}^{1/2}$$

The model coefficients $\hat{\beta}$ are given by the solution of the equation:

$$\sum_{i=1}^{K} \frac{\partial\mu_{i}^{\top}}{\partial\beta}V_{i}^{-1}\left( Y_{i}-\mu_{i} \right)=0$$

Parameters are typically obtained via the Newton-Raphson algorithm [38].

The variance and covariance structure is chosen to improve the efficiency of the parameter estimates. The algebraic form of the covariance matrix in the sample defines the variance structure. Examples of widely used working correlation specifications include independence, exchangeable, autoregressive, and unstructured form [24].

- The independence matrix assumes no correlation within clusters.

$$R_{i}=\left[ \begin{matrix} 1 & 0 & 0 & \cdots& 0 \\ 0 & 1 & 0 & \cdots& 0 \\ 0 & 0 & 1 & \cdots& 0 \\ 0 & 0 & 1 & \cdots& 0 \\ 0 & 0 & 0 & \cdots& 1 \end{matrix} \right]$$

- The exchangeable matrix assumes the same off-diagonal correlation value$\alpha$

$$\boldsymbol{R}_{i}=\left[ \begin{matrix} 1 & \alpha& \alpha& \cdots& \alpha\\ \alpha& 1 & \alpha& \cdots& \alpha\\ \alpha& \alpha& 1 & \cdots& \alpha\\ i & i & i & \cdots& \vdots\\ \alpha& \alpha& \alpha& \cdots& 1 \end{matrix} \right]$$

- The autoregressive matrix (AR) is based on an AR dependence across clusters.

$$R_{i}=\left[ \begin{matrix} 1 & \alpha& \alpha^{2} & \cdots& \alpha^{m-1} \\ \alpha& 1 & \alpha& \cdots& \alpha^{m-2} \\ \alpha^{2} & \alpha& 1 & \cdots& \alpha^{m-3} \\ \vdots& \vdots& \vdots& \ddots& \vdots\\ \alpha^{m-1} & \alpha^{m-2} & \alpha^{m-3} & \cdots& 1 \end{matrix} \right]$$

- The Unstructured correlation assumes symmetrically different correlation parameters.

$$\boldsymbol{R}_{i}=\left[ \begin{matrix} 1 & \alpha_{12} & \alpha_{13} & \cdots& \alpha_{1m} \\ \alpha_{21} & 1 & \alpha_{23} & \cdots& \alpha_{2m} \\ \alpha_{31} & \alpha_{32} & 1 & \cdots& \alpha_{3m} \\ \vdots& \vdots& \vdots& \cdots& \vdots\\ \alpha_{m1} & \alpha_{m2} & \alpha_{m3} & \cdots& 1 \end{matrix} \right]$$

# Model Residual Analysis

The residuals analysis (Figure S3) of the model has been performed by reporting the time series plot of residuals (Panel A), the Auto-Correlation Function (ACF) (Panel B), and the Histogram with the overlayed density (Panel C). The Box Pierce test for the autocorrelations of residuals has been also reported.

Figure S3 identifies the absence of a pattern in the residuals (Panel A). Some autocorrelations are slightly outside the bound (Panel B) for the first 10 lags however the Box Pierce test revealed the independence of the residual (p-value=0.97). Moreover, the histogram plots revealed the symmetry of residuals (Panel C). For the aforementioned reason, the residual analysis is deemed to be satisfactory.

# Computational details

The analysis has been conducted by using the R 4.2.1(2) System with the geepack(3) package.

The leading code function to obtain the model parameters is:

geese(hosp ~ ns(time,4),

sformula=~age, # covariate affecting overdispersion

id=id, # identification code for local district

data=agg.db,

family = "poisson",

corstr ="ar") #autoregressive structure for the cluster correlation

# Splines

The Smoothing spline (CIC = 32), the Restricted cubic spline (CIC=34); and the B-spline (CIC = 42) have also been considered for the computation. the Natural spline demonstrated a lower CIC value of 24.


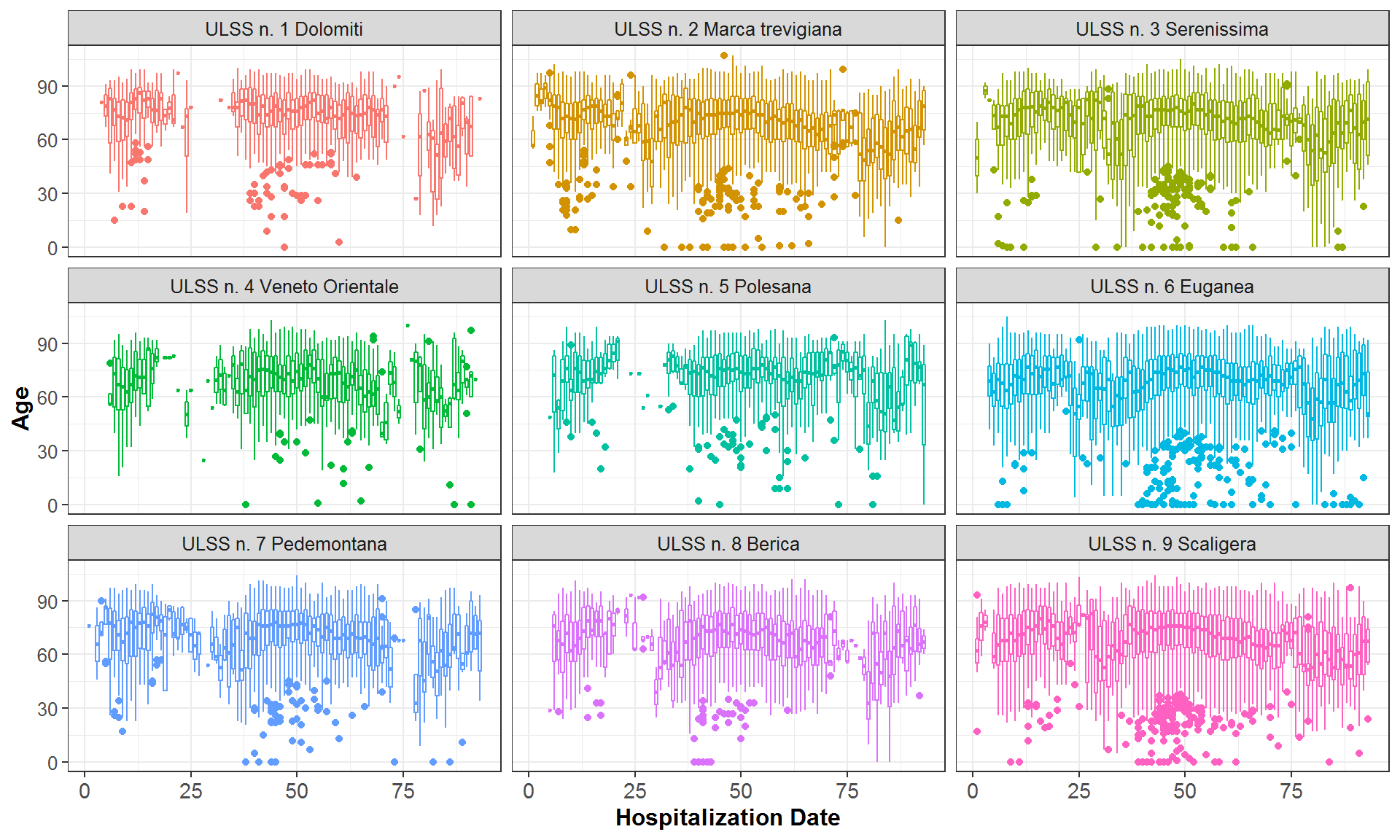


**Figure S1.** Age in hospitalization box plots according to admission date by local health district.


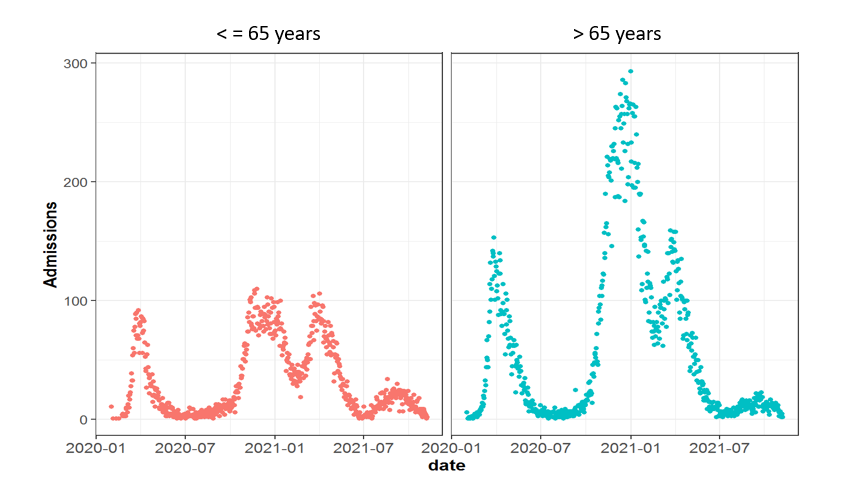


**Figure S2.** Numbers of hospitalizations according to age classes (<=65 years versus >65).


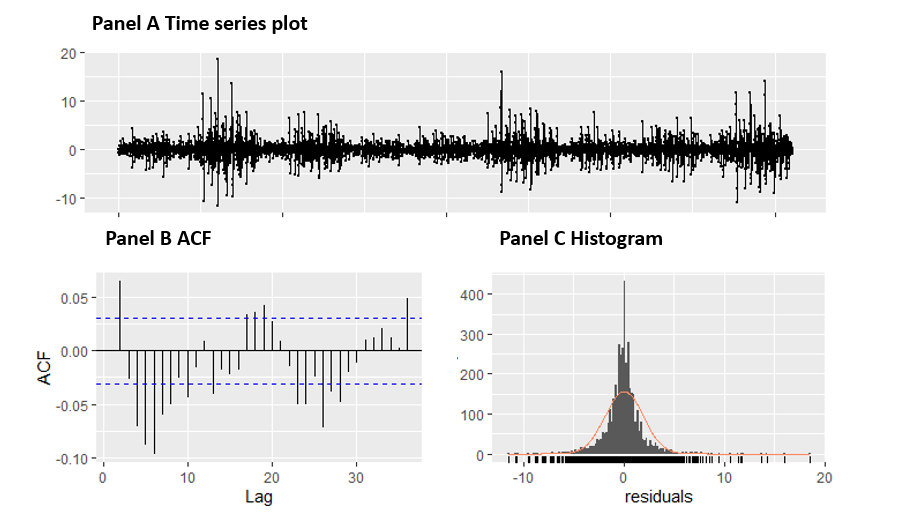


**Figure S3.** Residuals analysis (Figure S3) with the time series plot of residuals (Panel A), the Auto-Correlation Function (ACF) (Panel B), and the Histogram with the overlayed density (Panel C).

1. Zeger SL, Liang KY. Longitudinal data analysis for discrete and continuous outcomes. Biometrics. 1986;121–30.

2. R Core Team. R: A Language and Environment for Statistical Computing [Internet]. Vienna, Austria: R Foundation for Statistical Computing; 2022. Available from: https://www.R-project.org/

3. Halekoh U, Højsgaard S, Yan J. The R Package geepack for Generalized Estimating Equations. Journal of Statistical Software. 2006;15/2:1–11.
